# P-2081. Lessons Learned from Diverse Community Screenings and Client, Clinician, and Stakeholder Education to Improve HIV Detection in Priority Populations

**DOI:** 10.1093/ofid/ofaf695.2245

**Published:** 2026-01-11

**Authors:** Lesley Simon, Dean Beals, Stan Pogroszewski, Tabitha Washington, Rachel Deerr, Dovie L Watson, David P Serota, Jacqueline E Sherbuk, Charurut Somboonwith

**Affiliations:** dkbmed, Brooklyn, NY; DKBmed, Brooklyn, New York; DKBmed, Brooklyn, New York; DKBmed, Brooklyn, New York; DKBmed, LLC., Forest Hills, New York; University of Pennsylvania, Phiadelphia, Pennsylvania; University of Miami Miller School of Medicine, Miami, Florida; University of South Florida, Tampa, FL; USF Morsani College of Medicine, Tampa, Florida

## Abstract

**Background:**

In the United States, HIV disproportionately affects individuals in Southern states and those from historically marginalized groups, including people who are Black, formerly incarcerated, or facing food or housing insecurity. Community engagement is essential to ending the HIV epidemic in the US. Black men who have sex with men (MSM) are less likely to receive HIV testing in clinical settings than White MSM (65% vs 82%) and Black MSM are most likely to be tested in nonclinical settings (eg, HIV outreach program, syringe exchange program). The rate of new HIV diagnoses after testing in nonclinical settings is highest among Black individuals. We aimed to engage community stakeholders, implement community-based approaches to improving HIV detection in priority populations, educate healthcare clinicians, and identify lessons learned during the implementation of community-based approaches.

**Table 1.**
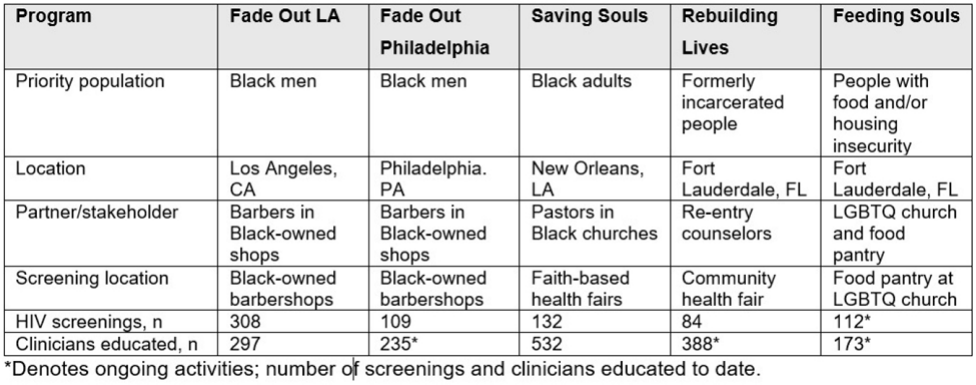
Program descriptions

**Figure:**
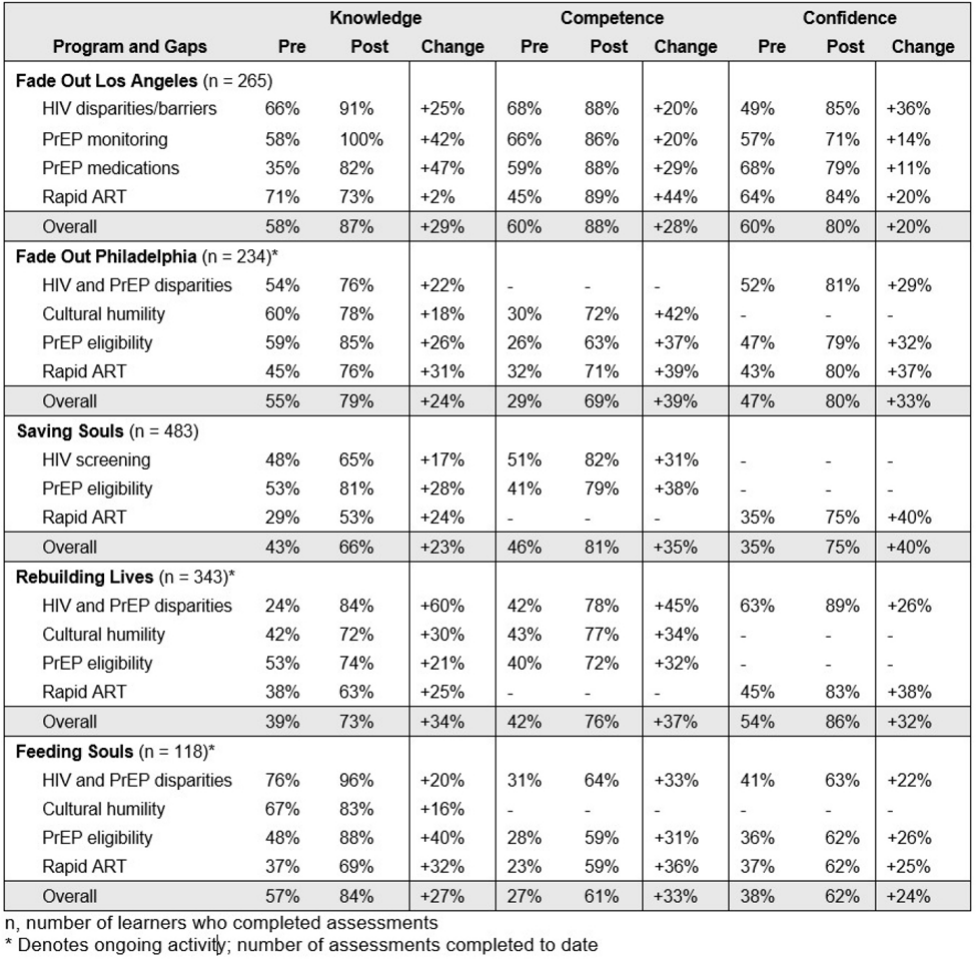

**Methods:**

We developed several multidimensional programs targeted towards priority populations identified in Ending the HIV Epidemic jurisdictions, each including three components: (1) accredited medical education for clinicians about the prevalence of HIV in the specific priority population and strategies to identify, prevent and treat HIV; (2) education for clients and partners/stakeholders related to that population; and (3) screening for HIV and other conditions at community events with incentives (eg, gift cards). Each programs’ components were determined in conjunction with stakeholders.

We assessed population and provider reach by collecting the number of people screened and number of clinicians who participated in the education, respectively. Educational outcomes were measured by assessing knowledge, confidence and competence before and after participation in the clinician education.

**Table 3.**
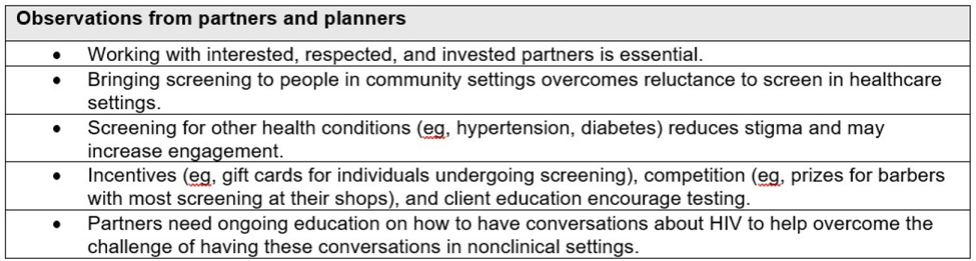
Lessons learned

**Results:**

To date, 1625 clinicians have been educated and 745 HIV screenings have been completed across five community events. See Table 1 for description of events, key populations, and stakeholders.

Educational outcomes are shown in Table 2.

Lessons learned, identified by the program planners and community partners, are shown in Table 3.

**Conclusion:**

Programs that incorporate client, clinician and partner education along with client incentives can promote HIV testing in priority populations.

**Disclosures:**

Lesley Simon, BA, Gilead Sciences: Independent medical education grant Dean Beals, BA, Gilead: CME Grants Stan Pogroszewski, JD, Gilead Sciences: Unrestricted CME Grant Tabitha Washington, MHA, Gilead Sciences, Inc.: independent medical education grant from Gilead Sciences Dovie L. Watson, MD, MSCE, Gilead Sciences: Honoraria

